# Full-length RNA structure prediction of the HIV-1 genome reveals a conserved core domain

**DOI:** 10.1093/nar/gkv1039

**Published:** 2015-10-17

**Authors:** Zsuzsanna Sükösd, Ebbe S. Andersen, Stefan E. Seemann, Mads Krogh Jensen, Mathias Hansen, Jan Gorodkin, Jørgen Kjems

**Affiliations:** 1 BiRC, Bioinformatics Research Centre, Aarhus University, DK-8000 Aarhus C, Denmark; 2 iNANO, Interdisciplinary Nanoscience Center, Aarhus University, DK-8000 Aarhus C, Denmark; 3 RTH, Center for non-coding RNA in Technology and Health, Department of Veterinary Clinical and Animal Sciences, University of Copenhagen, DK-1870 Frederiksberg C, Denmark

## Abstract

A distance constrained secondary structural model of the ≈10 kb RNA genome of the HIV-1 has been predicted but higher-order structures, involving long distance interactions, are currently unknown. We present the first global RNA secondary structure model for the HIV-1 genome, which integrates both comparative structure analysis and information from experimental data in a full-length prediction without distance constraints. Besides recovering known structural elements, we predict several novel structural elements that are conserved in HIV-1 evolution. Our results also indicate that the structure of the HIV-1 genome is highly variable in most regions, with a limited number of stable and conserved RNA secondary structures. Most interesting, a set of long distance interactions form a core organizing structure (COS) that organize the genome into three major structural domains. Despite overlapping protein-coding regions the COS is supported by a particular high frequency of compensatory base changes, suggesting functional importance for this element. This new structural element potentially organizes the whole genome into three major domains protruding from a conserved core structure with potential roles in replication and evolution for the virus.

## INTRODUCTION

The RNA genome of HIV-1 is situated in the viral capsid as two non-covalent linked positive stranded RNAs, with a length of ≈10 kb. It encodes several layers of information required for the viral replication cycle both for instructing protein synthesis and as functional RNA elements. The integrated provirus is flanked by long terminal repeats (LTRs), with a downstream 3′ region absent in the 5′ end of the RNA transcript, and an upstream 5′ region absent at the 3′ end of the RNA transcript. The viral genome contains structural (*gag*, *pol*, *env*), regulatory (*tat*, *rev*) and accessory (*nef*, *vif*, *vpr*, *vpu*) protein coding genes in up to three overlapping reading frames in the central part of the genome. HIV-1 is alternatively spliced, giving rise to proteomic diversity at different stages of its replication cycle.

The HIV-1 RNA transcript is 5′-capped, 3′-polyadenylated and contains several well-characterized RNA elements. The 5′ UTR of the transcript contains important structural signals for gene expression (1,2). The highly conserved transactivator region (TAR) hairpin, which recruits the viral Tat protein (3,4), is positioned at the 5′ end of the genome. This is followed by the *polyA-hairpin* containing a suppressed polyadenylation signal, but which plays an important role in nuclear export, dimerization and packaging (5). The primer binding site (PBS) stem exposes the PBS that binds the host cell tRNA^Lys3^. The packaging signal *Psi (Ψ)* stem contains the dimerization initiation site (DIS) in the distal loop that via a palindromic sequence mediates RNA–RNA interactions between the two RNA strands through the formation of a ‘kissing loop’. Next follows the splice donor (SD) stem, which contains the major 5′ splice site involved in generation of nearly all spliced viral mRNAs and a stem with the major packaging signal. Further downstream in the coding region, the *gag-pol frameshift* signal controls the ratio of Gag and Gag-Pol polyproteins, and a ‘slippery’ sequence in connection with an RNA stem is believed to be required for ribosomal frame-shifting. Towards the 3′ end of the genome in the Env coding region, the Rev response element (RRE) (6), composed of ≈350 nucleotides, forms a binding platform for multiple viral Rev proteins, enabling the nuclear export of the unspliced and singly spliced viral mRNA. A number of other structural studies have elucidated additional functional RNA structures in the HIV-1 genome, some of which have been reported to play roles in all aspects of the viral replication cycle, including modulation of ribosome processivity (7), alternative splicing (8), recombination-mediated gene swapping (9), protein evolution (10), dimerization (11,12) and circularization of the genome (13,14), as well as evading host defence mechanisms (15).

The structure of the HIV-1 5′ UTR has been studied for more than two decades both by chemical and enzymatic probing and bioinformatics and a consensus secondary structure including two long distance interactions have been derived (2,16–20).

More recently, a detailed secondary structure model has been proposed for the entire HIV-1 genome, based on the SHAPE technique (selective 2′-hydroxyl acylation analysed by primer extension) and a thermodynamic structure prediction algorithm (7). In the rest of this paper, we will refer to this as the Watts09 prediction model. The SHAPE technique probes the accessibility and flexibility of the 2′ hydroxyl groups in the RNA chain, and thereby detects the engagement of nucleotides in secondary and, to some extent, tertiary structures. This first complete structural model confirmed many previously suggested structures, but also featured a large number of previously uncharacterized secondary structures, which remain to be experimentally confirmed. The Watts09 model has been updated by modifying the parameters used to model SHAPE pseudoenergies (21), and more recently, based on the SHAPE-MaP technology, where mutational profiling is coupled to SHAPE-driven RNA structure prediction (22).

However, both the Watts09 structure and its updated version lack long-distance interactions and non-canonical base pairs. The prediction of such elements in the HIV-1 genome remains an open problem, which cannot be addressed by distance-constrained thermodynamic folding and SHAPE data alone. Instead, with the increasing number of HIV-1 sequences available, base pair co-variation in phylogenetic data can anchor RNA folding algorithms on biologically significant base pairs, and thereby greatly improve the quality of structure prediction for whole HIV-1 genomes.

In the present work, we apply a bioinformatics tool that combines a phylogenetic and a SHAPE data-driven approach (PPfold 3.1) to obtain a new HIV-1 secondary structure model without distance constraints (23,24). From our analysis, a view emerges that most of the HIV-1 genome appears relatively unstructured. Our results, therefore, generally support the hypothesis brought forward by Knoepfel and Berkhout (25) that HIV-1 does not belong to the group of viruses with a global genome-wide RNA structure (GORS) that for some RNA viruses has been proposed to be critical for virus replication (26,27).

However, we do identify several short-distance elements, which have yet to be experimentally confirmed, but which are supported by base pair covariations and are robustly predicted by a broad range of other bioinformatics methods. Importantly, our proposed structure also contains a distinct set of long-range base pairs supported by a significant number of clustered nucleotide covariations, which shape the genomic RNA into three major domains.

## MATERIALS AND METHODS

### Alignments

Alignments were downloaded from the Los Alamos HIV Sequence Database, http://www.hiv.lanl.gov/. For structure predictions, the latest (2010) HIV-1 subtype reference alignments for the ‘GENOME’ region were used, for HIV-1 strains A-K (group M, no recombinants). Strain G sequences are suspected recombinants (28) and were removed, because the evolutionary model in PPfold 3.1 does not describe recombination events. To enable comparison of our results with currently existing models, the alignment was extended with three strain B sequences: the sequence used in (7), the sequence used in (8) (accession number AF324493), and the GenBank reference sequence (accession number NC_001802). The alignment was lightly edited to correct any obvious misalignments. The resulting alignment consists of 38 sequences. The accession numbers of the sequences are listed in Supplementary Methods. For evaluating phylogenetic support for proposed structures, the complete 2011 HIV-1/SIVcpz web alignment (all sequences, including recombinants) containing 1850 sequences was used. To aid structural comparison, the sequence from (7) was aligned to it by hand. Subtype alignments were extracted from the two full genomic alignments directly, without manual curation. Alignments were edited in CLC Main Workbench 6 and MEGA 5.0 (29).

### SHAPE data

The SHAPE data for a complete HIV-1 genome used in this study had been previously published in (7). For training PPfold 3.1, the SHAPE data for *Escherichia coli* 16S and 23S rRNA sequences were used. These data were obtained from K. Weeks (personal communication).

### Structure predictions

Structures were predicted using several programs for RNA secondary structure prediction. For thermodynamic predictions based on free energy minimization, RNAstructure (30), UNAfold (31) and GTfold (32) were used. Each of them implements the nearest-neighbour thermodynamic model for RNA folding using the standard Turner free energy terms (33), but, due to slight implementation differences, the prediction results differ in less stable regions. RNAstructure and GTfold have integrated support for SHAPE data. Comparative predictions were done using RNAalifold (34,35), PPfold and PETfold (36,37). RNAalifold implements the nearest-neighbour thermodynamic model coupled to a model for converting covariation to pseudo-free energies. PPfold is based on a lightweight stochastic context-free grammar (SCFG), and is a re-implementation of Pfold with added support for probing data, such as SHAPE. PETfold integrates SCFG-based and thermodynamic predictions, but currently has no support for SHAPE data.

### Reliability scores

The formal definition and detailed discussion of the reliability scores is given in Supplementary Methods. On an empirical basis, we consider a score above 0.8 to be ‘high reliability’, a score between 0.5 and 0.8 to be ‘medium reliability’, and a score below 0.5 to be ‘low reliability’. High scores are only possible when the SHAPE data and evolutionary data support each other, and suggest a robust prediction. Low reliability scores are associated with random errors.

### Phylogenetic filtering of structures

Based on the SHAPE-directed PPfold 3.1 prediction (prediction number 4), covariations were computed based on the independent 1851-sequence alignment. For each predicted base pair, two-sided covariations and one-sided covariations (hemi-base pair covariations) were determined for the corresponding alignment columns in the following steps:

1. Sequences gapped in either column were discarded (in the consideration of that base pair only).
2. Of the remaining sequences in the alignment, the most frequently occurring base pair was identified.
3. Sequences with the most frequent base pair were discarded.
4. Canonical base pairs were counted among the remaining sequences, and classified into two-sided or only one-sided covariations with respect to the most frequent base pair.
5. One-sided and two-sided covariations were expressed as a% of the number of possible covariations.

Prediction results were then filtered by removing base pairs that fulfilled at least one of the following conditions:

1. The number of possible covariations was less than 5% of the number of sequences. This was to make sure a sufficient number of sequences were available.
2. One-sided covariation is less than 75% AND two-sided covariation is less than 25%. (Here, two-sided covariations are considered to be a subset of one-sided covariations.)

### Data processing

Data processing and analysis was done in MATLAB R2010b. Covariation was evaluated by counting compensating base changes in alignments.

### Structure drawings

Structure drawings were made in XRNA 1.1.12 and JViz.RNA.

### Data access

The prediction results for all predictions are included in Supplementary Materials (Supplementary Table S1).

## RESULTS

### A comparative model for the higher-order structure of the HIV-1 genome

**Figure 1.**
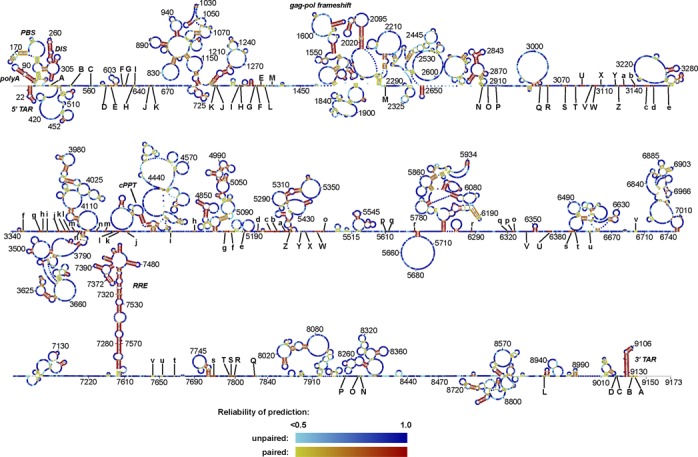
A comparative model for the secondary structure of the HIV-1 genome, predicted with PPfold 3.1, integrating covariation information from a manually curated alignment of 38 HIV-1 genome sequences and structure probing data from the SHAPE method. Each nucleotide is coloured according to the reliability score (described in Materials and Methods). Long-distance interactions (further than 600 nucleotides apart) are indicated with letter codes. Corresponding capital and non-capital letters interact. A higher resolution version of the figure with nucleotide identities is available in Supplementary Figure S2.

**Figure 2.**
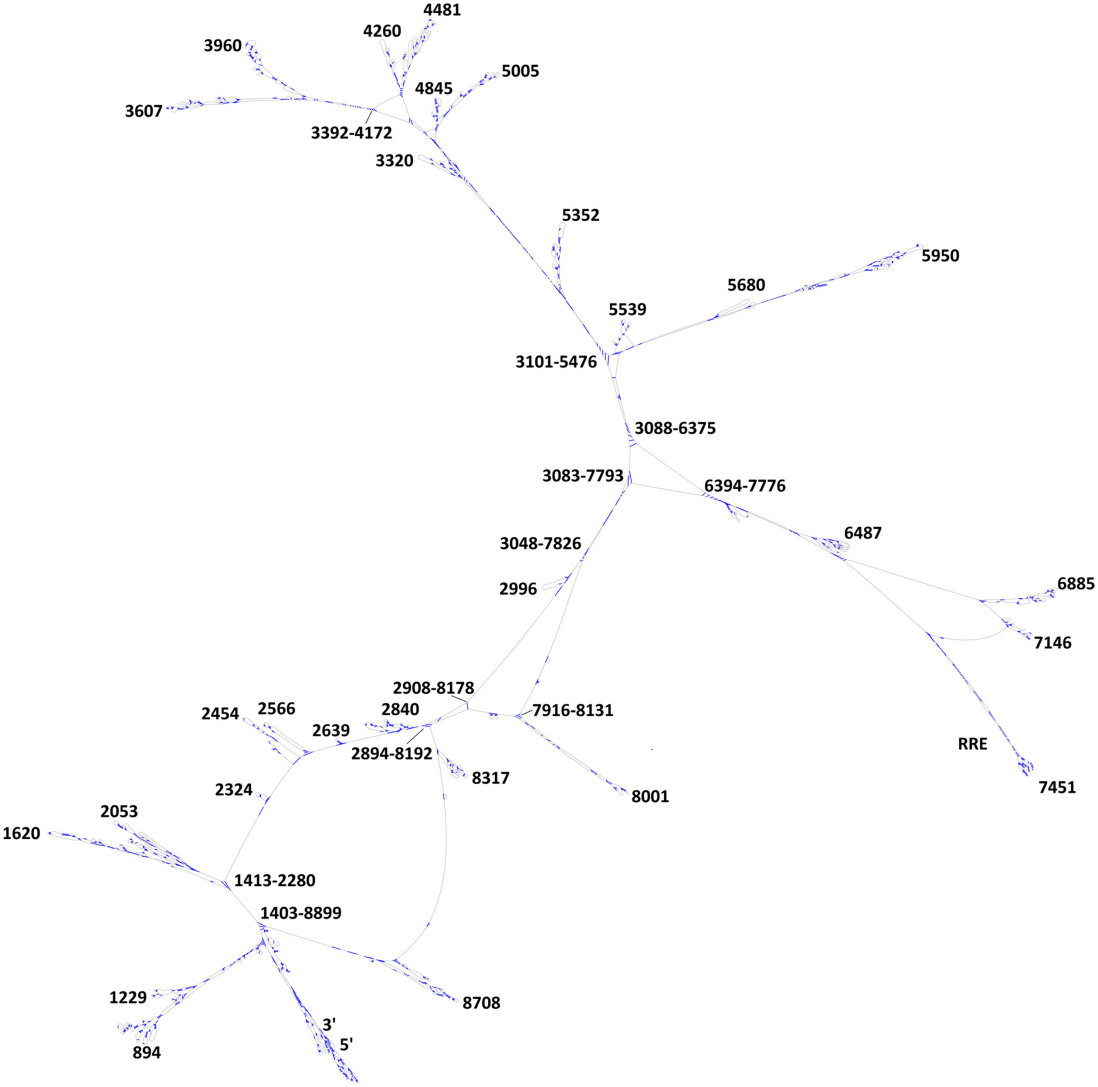
Topology of the predicted consensus secondary structure of the HIV-1 genome, predicted with PPfold 3.1. Base pairing is indicated by blue lines. The structure was drawn in jViz.Rna (50) and annotated manually after visual inspection.

We predicted the consensus secondary structure of an HIV-1 alignment using PPfold 3.1, which is a re-implementation of the Pfold algorithm (38) extended with probabilistic support for RNA probing data (23). As input, we used a manually curated alignment of sequences from HIV-1 subtypes A-K (excluding strain G; see Materials and Methods), and the experimental SHAPE data from (7). In order to maximize useful phylogenetic information and minimize noise (39), we predicted the HIV-1 structure from 38 representative sequences, as described in Materials and Methods (Figures 1 and 2). A larger alignment of 1851 HIV-1 sequences was then used as an independent evaluation of the phylogenetic evidence for the predicted structures.

In the prediction by PPfold 3.1, the evolutionary covariation information derived from the alignment was weighted equally with the SHAPE data measured for one B-strain sequence, integrating the available data for the best secondary structure model for this particular sequence. Therefore, the consensus structure prediction does not necessarily correspond to the optimal structure of each individual strain, and our result is biased towards the optimal structure for the B-strain sequence for which the SHAPE data were available.

Our approach is different from that described in (7) and (22) in a number of ways. No restriction is introduced on base pairing contact distance, enabling the prediction of long-distance interactions across the entire genome. Non-canonical base pairs are also predicted, as the evolutionary model implemented in PPfold 3.1 supports non-canonical covariance. Finally, the prediction is based on a model that integrates evolutionary and experimental information in the probabilistic framework of a stochastic context-free grammar, rather than a thermodynamic model that only uses information from SHAPE data. We have previously shown that the performance of the PPfold 3.1 algorithm on large ribosomal data sets is comparable to RNAstructure (30) for single-sequence SHAPE-directed predictions, and superior in the case of moderately good alignments (23). Our structural model for the entire HIV-1 genome complements the Watts09 model, and adds a new layer of information about long distance interactions and non-canonical base pairs.

### Comparison of the PPfold 3.1 and Watts09 predictions

**Figure 3.**
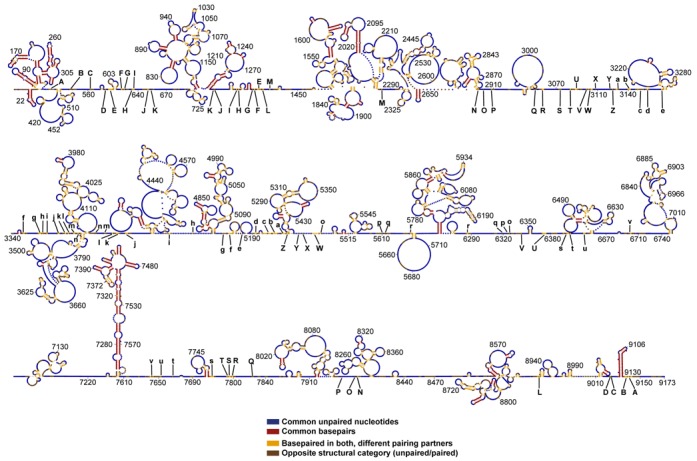
The PPfold 3.1 structure coloured by comparison with the Watts09 structure. The predictions were made with identical SHAPE data. Common structural elements are indicated in red (base pairs) and blue (unpaired nucleotides). Elements paired in both structures (but with different partners) are indicated by yellow. Nucleotides paired in one but unpaired in the other are indicated in brown. 72% of all unpaired nucleotides and 31% of base paired nucleotides were found in both predictions. Over half of the genome is unpaired in both predictions (blue). A higher resolution version of the figure with nucleotide identities is available in Supplementary Figure S3.

The structures predicted by PPfold 3.1 and Watts09 were compared and similarities and differences are colour coded in Figure 3. It is clear that several short-distance structural elements are shared between the Watts09 model and our prediction (red helices and blue single stranded regions). However, it is also immediately apparent that our structure is significantly different from the Watts09 structure, with only 31% of the base pairs (92 helices) in common. As both predictions are based in part on the same SHAPE data, an assessment of the differences requires additional analysis.

Both predictions are affected by random errors due to the well-known lack of robustness of RNA structure prediction (40). In addition, they are based on different statistical models, so differences can arise even in regions that are otherwise robustly predicted. To distinguish these systematic differences from the random uncertainties, we assessed the reliability scores reported by PPfold 3.1. (Figure 1, and Supplementary Figure S1, top) The reliability score for a structure prediction at a nucleotide is the sum of the probabilities of all structures containing that structure prediction, under the statistical model implemented in PPfold 3.1. The statistical model combines a stochastic context-free grammar with likelihoods from phylogenetic and SHAPE data. (For more information, see Materials and Methods and Supplementary Methods). High reliability scores (0.8 or greater) indicate robustness, and are only possible when the SHAPE data and the phylogenetic data are consistent with each other. Therefore, structural conflicts at high-reliability nucleotides are likely to be caused by systematic differences between the two prediction methods, rather than random errors in the PPfold prediction.

We expected that the common structures in the Watts09 and PPfold 3.1 predictions would generally have high reliability scores, and this is also what we observed, both for paired and unpaired nucleotides. Interestingly, more than half of all nucleotides of the HIV-1 genome remained unpaired in both structures, and the vast majority of these nucleotides also had a high reliability score (Figures 1 and 3 and Supplementary Figure S1). This is evidence of less base pairing in the HIV-1 genome than cellular structural RNAs, such as ribosomal RNA, where typically less than 40% of the structure is single-stranded. (41,42)

Most of the common base pairs are part of already known structures, including the TAR hairpin and other parts of the HIV-1 leader sequence, the *gag-pol* frameshift hairpin element, the RRE and the 3′ TAR hairpin (Figure 3). These structures also had generally high reliability scores, confirming that they are supported by phylogeny as well as the probing data. In addition to this, a number of smaller stems scattered throughout the genome were present in both predictions, but we could not confirm any of the other extensive local structures proposed in the Watts09 prediction.

Of nucleotides with conflicting structures in the two predictions, nearly half were paired in the Watts09 structure, while being unpaired in the PPfold 3.1 structure (Supplementary Figure S1). The majority of these nucleotides also had a high reliability score, suggesting that an important reason for finding conflicting structures in these positions was a different interpretation of the SHAPE values. Indeed, the median SHAPE reactivity was 0.2 for nucleotides in this group, and it has recently been shown (43) that the pseudo-energy terms used in the Watts09 prediction significantly overestimate the base pairing likelihood for SHAPE values of this magnitude. The likelihood-based model implemented in PPfold 3.1 considers these bases more likely to be unpaired. According to (43), SHAPE values of this magnitude are more consistent with unpaired state as suggested by PPfold 3.1, rather than the paired state as suggested by the Watts09 prediction. This further increases our confidence that the majority of the HIV-1 genome is significantly less structured than structural RNAs.

An interesting class of differences arise where both predictions contain base pairing, but the pairing partners are different. Of these nucleotides, 36% had a high reliability (Supplementary Figure S1), indicating that phylogenetic support was used by PPfold 3.1 to resolve the base pairing partners. Interestingly, of the high-reliability base pairs where the base pairing partners are different, 22% are long-distance (>600 nt contact distance, 89 nucleotides). We describe these in more detail in the next section.

Only 8% of all nucleotides were base paired in the PPfold 3.1 prediction, while staying unpaired in the Watts09 prediction. The median SHAPE value for these nucleotides was 0.3 (Supplementary Figure S1), which signals a moderately high degree of flexibility. This group of nucleotides accounts for over 56% of the non-canonical interactions predicted by PPfold 3.1, and might contain unusually flexible base pairs or tertiary interactions.

In a recent study by Pollom *et al*. (21), the same HIV-1 sequence was re-folded using slightly modified parameter values in the same thermodynamic prediction method. The Pollom 2013 prediction (Pollom13) contained 351 extra base pairs (an increase of 19%) compared to the Watts09 prediction, because the revised pseudoenergies increased the base pairing likelihood for high SHAPE values even further than in the Watts09 prediction. Despite this, only 68% of the base pairs found in the Watts09 prediction were also preserved in the Pollom13 prediction (see Venn diagram in Supplementary Figure S7). Notably, a very large percentage (373/429 = 87%) of the base pairs that are common between the Watts09 and PPfold 3.1 predictions are also found in Pollom13. Hence, we can conclude that the overlap between the Watts09 and PPfold 3.1 predictions is very similar to the overlap between the Pollom13 and PPfold 3.1 predictions.

### Long-distance interactions in the HIV-1 genome

**Figure 4.**
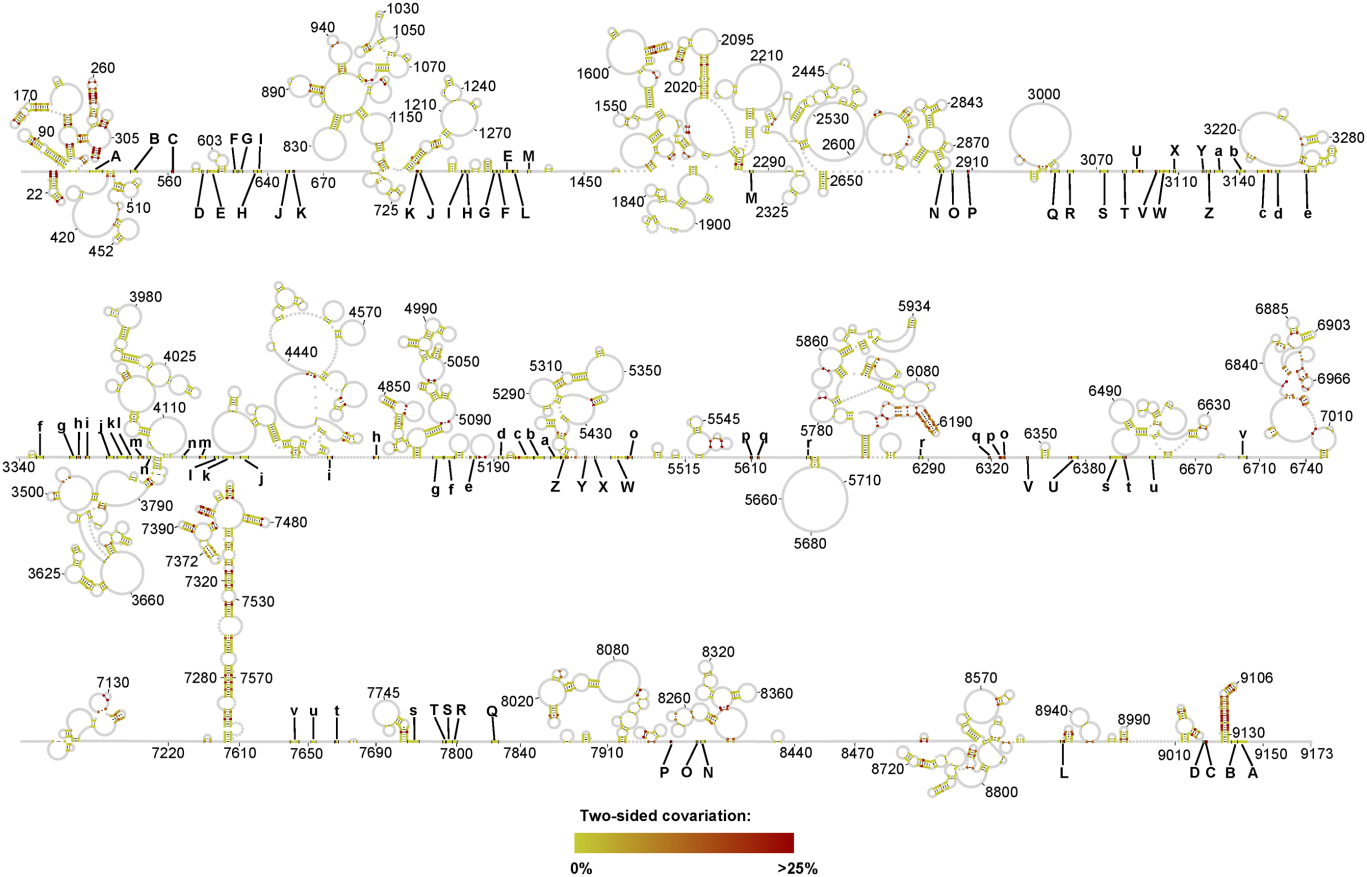
Phylogenetic analysis of the proposed structural model. The proportion of base pair covariations (two-sided covariation) is plotted. This is the proportion of base pairs consistent with the structural prediction (A-U, G-C, G-U), when removing gaps and the most frequent canonical base pair, as described in the Materials and Methods section. A higher resolution version of the figure with nucleotide identities is available in Supplementary Figure S4.

A particular challenge in the phylogenetic analysis of HIV-1 is the high degree of sequence conservation in the ge and 3 distant regionsnome (Supplementary Figure S5). The median percentage of conservation across all columns of the 1851-genome alignment was 96%. Due to the high degree of conservation, base pair covariations are sparse (Figure 4) and most frequent in helices within the well-known 5′ leader, the RRE and the 3′ TAR regions.

**Figure 5.**
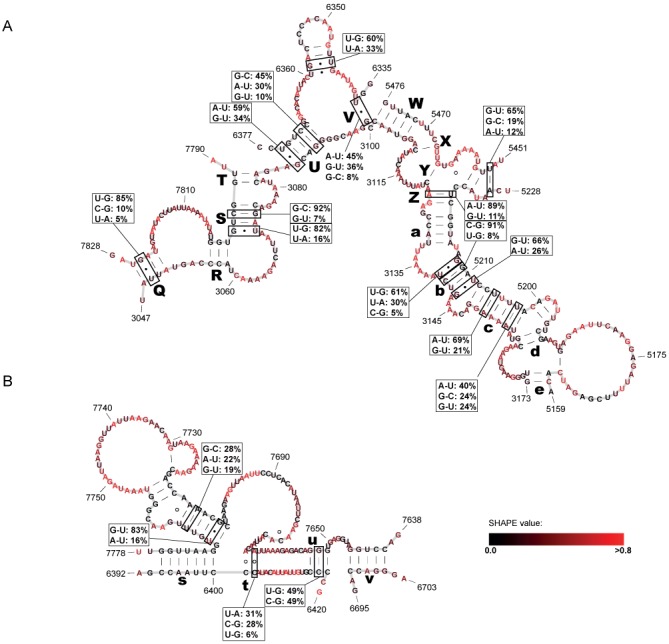
Putative long-distance interactions in the HIV-1 genome. (**A**) Core organizing structure (COS) in the central domain of the HIV-1 genome, coordinating the formation of three arms. (**B**) Putative long-distance interactions in the ‘RRE domain’ of the HIV-1 genome. The letters correspond to those in Figure 1. Base pairs with significant base pair covariations are indicated, in the 5′-3′ direction.

Interestingly, the highest incidence of base pair covariations outside these well-defined structural elements confines a set of long-range interactions. Most prominent are the interactions that involve on one side of a stretch of ≈107 nucleotides (positions ≈3048–3155) and on the other side 3 distant regions: 5192–5219, 6337–6375 and 7790–7826, counting a total of 12 base pair covariations (Figure 5 (A)).

This set of long-range interactions that we coin ‘core organizing structure’ (COS) defines an overall topology of our predicted HIV-1 B-type structure that features three ‘arms’. In this model, important structural elements, such as the 5′ leader sequence and the RRE, are exposed in the distal parts in two of the arms (Figure 2). Additional high-reliability long-distance interactions are predicted in the region 6390–7780, forming the ‘RRE arm’ of the topology. Many of these interactions are also strongly supported by covariation evidence (Figure 5 (B)).

### Strong short-range secondary structure signals in HIV-1

Despite the evidence of a low degree of stable base pairing in the HIV-1 genome, a number of local RNA secondary structures are highly conserved and may play important biological roles. To detect the strongest structural signals for short-range interactions, we identified the base pairs that were both robustly predicted and exhibited base pair covariations, as follows. Firstly, we predicted the secondary structure of the HIV-1 genome using a wide range of programs and parameter setups (Table 1). One of the predictions (prediction 13) was made with PPfold 3.1 using re-normalized SHAPE data, to account for the fact that the percentage of nucleotides with normalized SHAPE reactivities over 1.0 was 9% in the case of the ribosomal data set used to train the thermodynamic models, but only 5% in the case of HIV-1. Detailed information about this re-normalization procedure is provided in Supplementary Methods.

**Table 1.** Summary of secondary structure predictions

Prediction number	Software	Method	Comparative	SHAPE data	Max. contact distance = 600 nt
1	RNAstructure	Thermod.	NO	YES	YES
2	RNAstructure	Thermod.	NO	NO	YES
3	PPfold 3.1	SCFG	YES	YES	NO
4	PPfold 3.1	SCFG	YES	NO	NO
5	GTfold (Aug 2011)	Thermod.	NO	NO	NO
6	GTfold (Aug 2011)	Thermod.	NO	YES	NO
7	GTfold (Aug 2011)	Thermod.	NO	NO	YES
8	GTfold (Aug 2011)	Thermod.	NO	YES	YES
9	UNAfold	Thermod.	NO	NO	NO
10	UNAfold	Thermod.	NO	NO	YES
11	PETfold	Combin.	YES	NO	NO
12	RNAalifold	Thermod.	YES	NO	NO
13	PPfold 3.1	SCFG	YES	YES (re-normalized)	NO

Prediction 1 is identical to the structure published in (7). In SHAPE-driven predictions, unless otherwise indicated, the SHAPE data published in (7) were used without re-normalization. The details for re-normalization used for prediction 13 are described in Supplementary Methods. In phylogenetic predictions, the manually curated HIV-1 alignment, described in Materials and Methods, was used. The full pairing schemes are provided in Supplementary Table S1.

**Figure 6.**
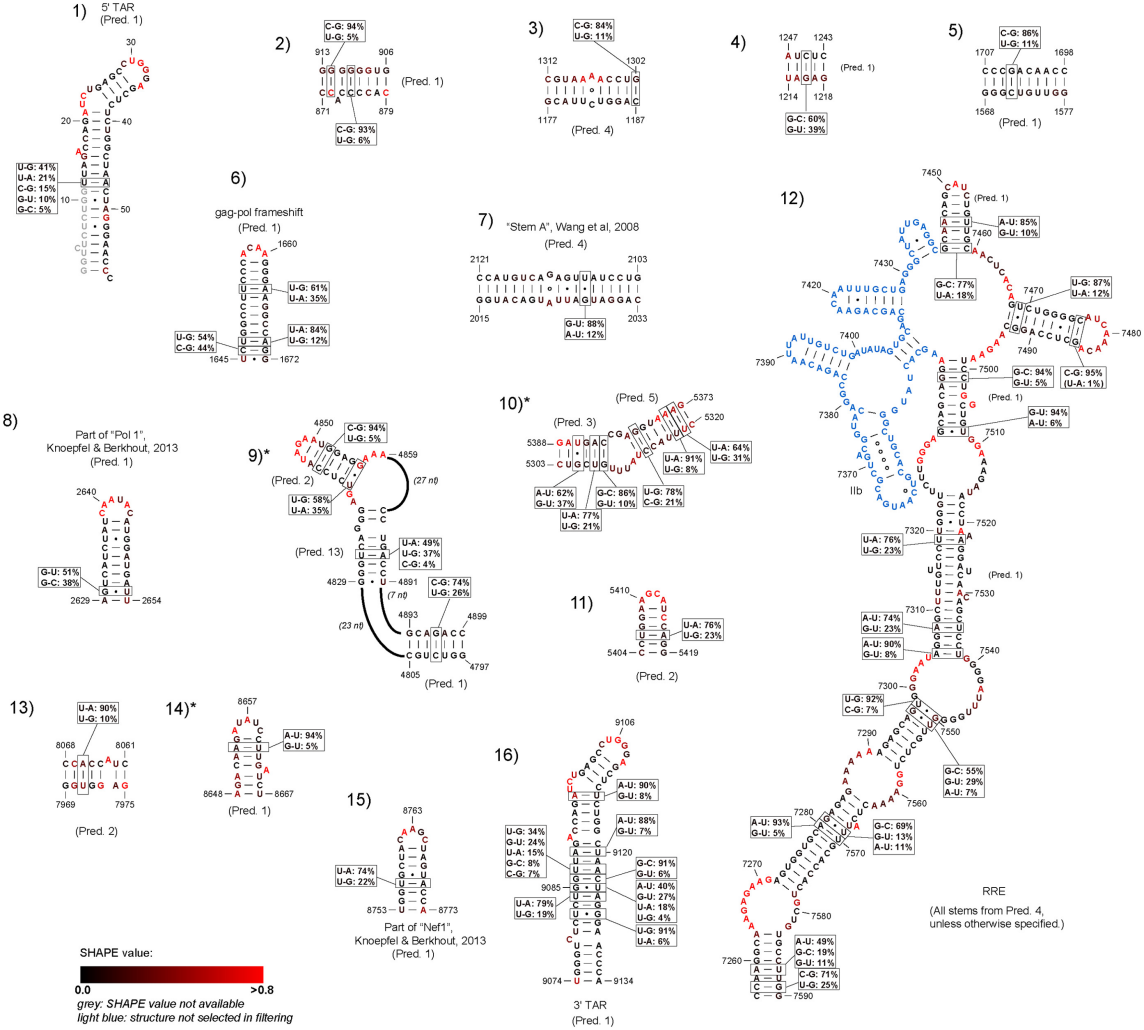
Short-distance interactions supported both by consistency of prediction and covariation. Base pair covariations are indicated as boxes for qualifying base pairs. SHAPE values are indicated on a scale from black (low degree of flexibility) to red (high degree of flexibility). Missing SHAPE values at the start of the 5′ TAR hairpin are indicated in grey. The part of the RRE structure that did not pass the stringent filtering criteria is indicated in light blue. The structures that were not present in the most recent update of the Watts09 structure (22) are indicated with asterisk (*). Structure 9 is partially present in (22).

Next, we identified the base pairs in the PPfold 3.1 prediction that were also present in at least 8 of the other 12 predictions. As expected, few structures were consistently predicted at this level of stringency: only 290 base pairs in 57 helices were present in at least 8 other predictions. To identify the structural elements that were also supported by base pair covariations, we further filtered the 57 helices according to the criteria described in the Materials and Methods section. After this filtering, 27 helices in 16 structural domains remained. The longest version of each helix was selected across all predictions, and these high-confidence substructures are depicted in Figure 6.

Of the known structural elements, the 5′ TAR hairpin (structure 1), the gag-pol frameshift element (structure 6), a large part of the RRE (structure 12) and the 3′ TAR hairpin (structure 16) were all consistently predicted and supported by extensive covariation evidence. Notably, stem-loop IIB (7351–7378) in the RRE, which constitutes the high-affinity binding site for Rev, is inconsistently predicted, because of the presence of extensive non-canonical pairings in this region. The SHAPE-driven PPfold 3.1 (prediction 4) predicted two C-U (U7354-C7373, C7356-U7370), one G-G (G7355-G7371) and one A-C (A7357-C7369) base pairs in the region. These non-canonical pairings were predicted by the other comparative setups as well, but not by the purely thermodynamic methods. However, none of the methods predicted the critical non-canonical base pairs determined by X-ray crystallography (the invariant G7351-A7377 and G7352-G7375 (44)), which are required for Rev recognition. This is presumably a result of 100% sequence conservation in the alignment columns involved in these thermodynamically unusual base pairs.

Most of the remaining structures in our Figure 6 are also confirmed in other studies. In particular, structures 8 and 15 overlap with the ‘Motif E/POL1’ and ‘Motif O/NEF1’ structures studied in a recent experimental work. (25) Both of these structures were also among the highest-confidence stems in a bootstrapping analysis performed by Kladwang *et al*. (45); bootstrapping probabilities at 100% and 98.5% for these structures, respectively. Furthermore, both of them were present in the recent update of the HIV-1 secondary structure using SHAPE-Map technology (22).

Structure 7 has previously been proposed to form in the coding region of the pol gene, and may play a role in recombination (46). In that study, Wang *et al*. suggested a more complex structure formed by three stems (A, B and C); however, only stem A passed our stringent filtering. Stem B was present in all predictions, but lacked base pair covariations, whereas stem C was inconsistently predicted. Structure 7 was also present in both the SHAPE-Map work (22) and among the highest-confidence stems described in (45), with a bootstrapping probability of 99.5%.

The remaining consistently predicted and evolutionarily supported stems have no known function to our knowledge, and any biological role for them remains to be studied experimentally. However, several of them have been confirmed in (22) and (45). Structures 4 and 5 were confirmed in both. Structures 9 and 11 had high confidence in (45) but was not reported in (22). Structure 3 was reported in (22), but had low confidence in (45). Structure 13 was also reported in (22), but was not present in (45). The least reliable structures in our Figure 6 are 2, 10 and 14, which had both low bootstrap confidence values in (45), and were missing in (22).

Altogether, these results show that the already well-characterized elements remain among the strongest structural signals in the HIV-1 genome.

## DISCUSSION

We have employed a novel bioinformatics tool, PPfold 3.1, that combines phylogenetic and a SHAPE data-driven approaches to predict a new HIV-1 secondary structure model without distance constrains. The result provided information about the structures both on a global scale as well as on a single nucleotide level. On the global scale we found that an unusually low degree of base pairing in the HIV-1 structure (over 60% unpaired nucleotides). This result is intriguing, and it is important to ask if it is an artefact of the prediction method, or if there is evidence that HIV-1 genomic RNA is significantly more flexible than structural RNAs. Our results show that both experimental SHAPE data and a wide range of secondary structure prediction methods support the picture of a highly flexible HIV-1 genome structure, with relatively few stable RNA secondary structures forming compared to known naturally structured RNA. This conclusion is in line with previous speculation that the HIV-1 retrovirus does not belong to the group of RNA viruses with GORS (25).

There may be several biological explanations for this. Most of the genome encodes proteins, even from up to three overlapping reading frames, and also displays a complex set of splicing regulatory sequences and functionally important binding sites for viral and cellular proteins. The combination of these elements puts strong evolutionary pressures on the RNA, which may not be compatible with the development of extensive stable secondary structures.

It has also been suggested that the dsRNA-specific recognition by the host RNA interfering system can be evaded by avoiding longer stable RNA structures (7). Also, the RNA needs to be reverse transcribed into DNA, and stable hairpins are known to inhibit the processivity of the reverse transcriptase. Finally, the folding of the HIV-1 genome is also facilitated *in vivo* by viral proteins, most importantly the nucleocapsid (NC) protein, which possesses ATP-independent RNA chaperone activity by binding nonspecifically to the RNA structure. As NC is found in extremely high concentrations in the virions, it may play an important role in protecting of single stranded regions and stabilizing less stable RNA duplexes in the HIV-1 genome. Curiously, the nucleotide composition of HIV-1 is heavily biased towards A's (47,48), which have a higher tendency to be unstructured, a phenomenon also observed in the ribosome (where 66% of A's are unpaired). It is possible that HIV-1 exhibits a preference for A's in order to retain a largely unstructured, flexible genome.

Our prediction was not compatible with many of the short-range secondary structure elements suggested in the Watts09 model, with just over 31% of the base pairs in common, even though both predictions are consistent with the originally published SHAPE data. For completeness, we note also that the recent update of the Watts09 structure (22) also only shares 56% of the base pairs with the original Watts09 prediction, despite the two predictions were based on very similar bioinformatics principles—and the PPfold 3.1 structure presented in this paper only shares 29% of the base pairs with the updated Watts09 structure. The large number of significantly different structures obtained for the HIV-1 genome with very similar methods reflects a lack of robustness in RNA secondary structure prediction. This lack of robustness is a critical factor to consider even in the SHAPE-directed case, and it highlights the need for additional studies to confirm the proposed structures. Indeed, a recent study by Knoepfel and Berkhout (25) mutated versions of any of 16 selected stems from the Watts09 model showed no significant replication defects in an ultra-sensitive virus competition assay upon extended culturing (25). Two of the structures also appear in our list of consistently predicted structures (structures 8 and 15). It remains to be studied whether our additional predicted structures can be experimentally supported.

Our predicted folding of the HIV-1 5′ UTR (1–336) is largely in agreement with the Watts09 model and other phylogenetic predicted secondary structures as referred to in the introduction (Note that the TAR is most likely incorrectly in our model due to the lack of SHAPE data of the initial 11 nucleotides). However, down stream from the 5′ UTR (343–750) the two models completely divert. This is most likely a consequence of our predicted long distance interactions (B-K; Figure 3) located in the 540–660 region. Notably, our model does not predict the long-range interaction formed between the 79–85 and 443–449 regions, suggested by Paillart et al., presumably because the both sequences are highly conserved in HIV-1.

Perhaps the most significant result from our study is the finding of phylogenetically supported long distance interactions (the COS) that have the potential to organize the entire genome into three subdomains. Interestingly, part of the core coincides with a region shown to constitute a recombination hotspot in vivo (49). In that study, a different local secondary structure was suggested for nucleotides 6396–6513, based on a local folding of the RNA, which conflicts with the long-distance interaction proposed here. The potential role of the COS in recombination remains to be elucidated.

Interestingly, the COS organizes the entire genome into three major domains. One of the domains may facilitate exposure of the RRE to the Rev protein, another domain the DIS for genome dimerization and PSI for Gag recognition in connection with viral packaging. A third domain, which mainly constitutes the Pol gene have not yet been assigned any role at the RNA level but the prominent RNA secondary structures predicted between 3400 and 5100 may also protrude from the core structure and play a, yet unknown, functional role.

## Supplementary Material

SUPPLEMENTARY DATA
