# Supplementary material for: Full-length RNA structure prediction of the HIV-1 genome reveals a conserved core domain
Source: Nucleic Acids Res. 2015 Oct 17;43(21):10168–79. doi: 10.1093/nar/gkv1039 (PMC4666355; doi:10.1093/nar/gkv1039)
Supplement: SUPPLEMENTARY DATA [file supp_43_21_10168__index.html]

Full-length RNA structure prediction of the HIV-1 genome reveals a conserved core domain — SUPPLEMENTARY DATA 

# Full-length RNA structure prediction of the HIV-1 genome reveals a conserved core domain

## SUPPLEMENTARY DATA

- SUPPLEMENTARY DATA
- SUPPLEMENTARY DATA
- SUPPLEMENTARY DATA
