# Supplementary material for: Full-length RNA structure prediction of the HIV-1 genome reveals a conserved core domain
Source: Nucleic Acids Res. 2015 Oct 17;43(21):10168–79. doi: 10.1093/nar/gkv1039 (PMC4666355; doi:10.1093/nar/gkv1039)
Supplement: SUPPLEMENTARY DATA [file supp_gkv1039_nar-01265-r-2015-File008.pdf]

Supplementary Figures

Supplementary Figure S1: Illustration of the grouping of the nucleotides in the HIV-1 genome by structural category (top row), and boxplots of the PPfold 3.1 reliability scores and SHAPE values for each structural category (bottom row). (A) nucleotides that were unpaired in both structures (B) shared base pairs (C) nucleotides that were paired in the PPfold 3.1 structure, but unpaired in the Watts09 structure (D) nucleotides that were paired in both structures, but did not share pairing partners (E) nucleotides that were paired in the PPfold 3.1 structure, and unpaired in the Watts09 structure.

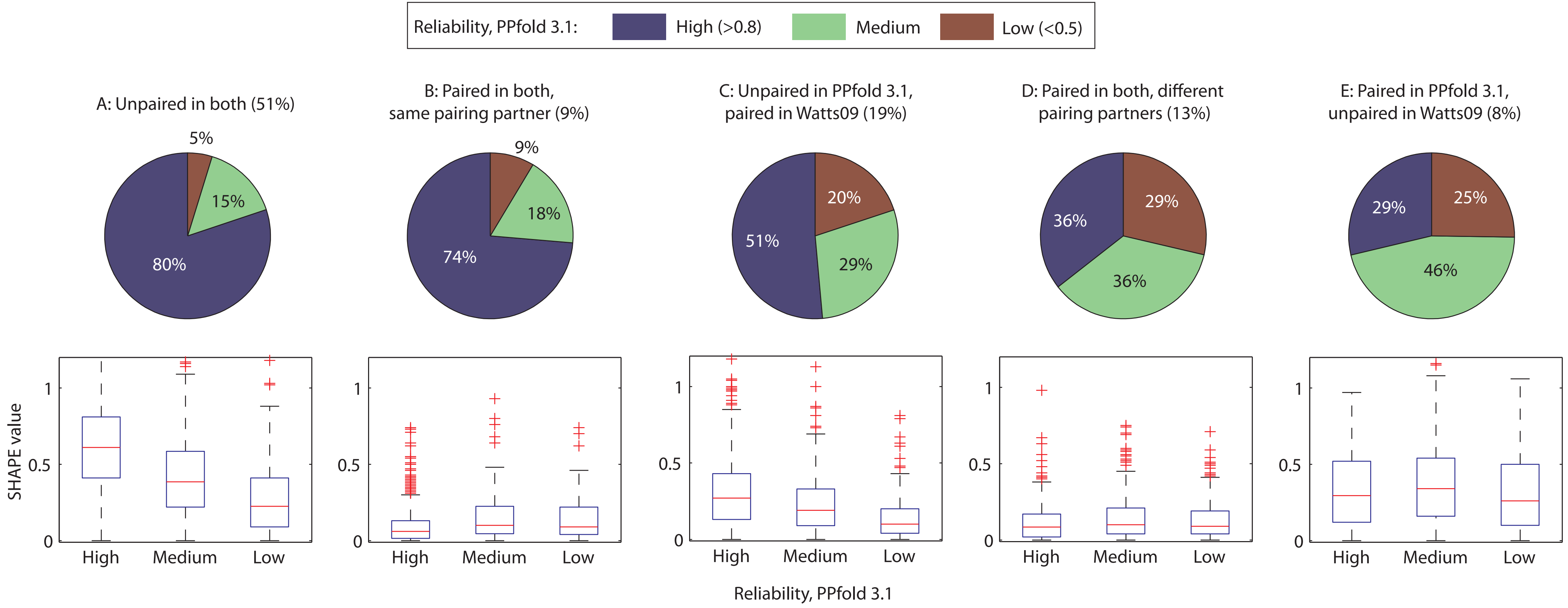

Reliability, PPfold 3.1:
High (>0.8)
Medium
Low (<0.5)
A: Unpaired in both (51%)
5%
15%
80%
B: Paired in both, same pairing partner (9%)
9%
18%
74%
C: Unpaired in PPfold 3.1, paired in Watts09 (19%)
20%
29%
51%
D: Paired in both, different pairing partners (13%)
29%
36%
36%
E: Paired in PPfold 3.1, unpaired in Watts09 (8%)
25%
29%
46%
SHAPE value
0
0.5
1
High
Medium
Low
Reliability, PPfold 3.1

Supplementary Figure S2: comparative model for the secondary structure of the HIV-1 genome, predicted with PPfold 3.1, integrating covariation information from a manually curated alignment of 38 HIV-1 genome sequences and structure probing data from the SHAPE method. Each nucleotide is coloured according to the reliability score (described in Methods). Long-distance interactions (further than 600 nucleotides apart) are indicated with letter codes.

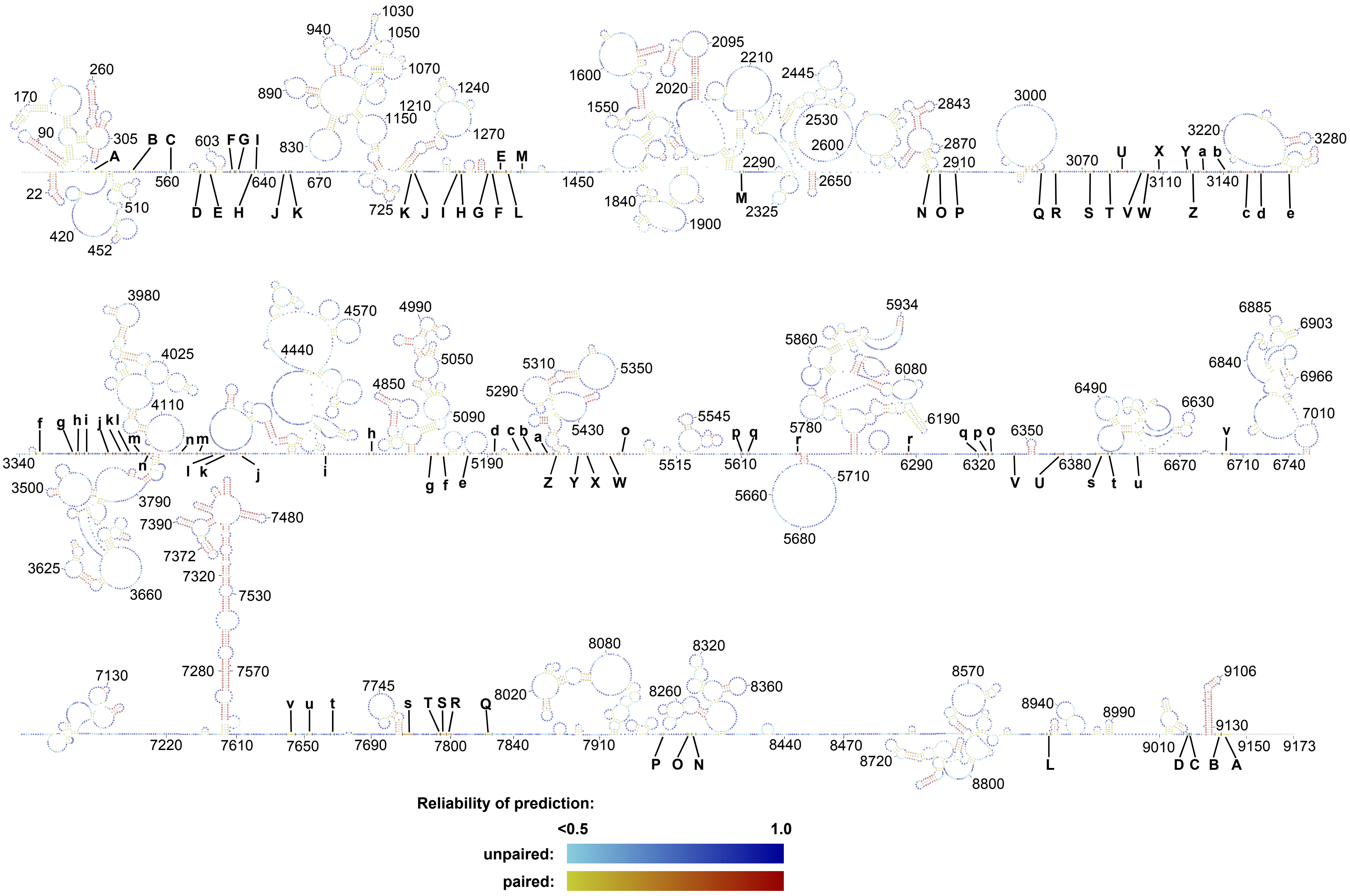

22
90
170
260
305
420
452
510
A
B
C
560
603
F G I
D
E
H
640
J
K
670
830
890
940
1030
1050
1070
1150
1210
725
1240
1270
K J I H G F L
E
M
1450
1550
1600
1840
1900
2020
2095
2210
2290
M
2325
2445
2530
2600
2650
2843
2870
2910
N O P
3000
Q R
3070
S T V W
U
X
Y a b
3110
3140
Z
c d e
3220
3280
3340
f g h i j k l m
n m
n
l k
j
i
3500
3790
3625
3660
3980
4025
4110
4440
4570
4850
4990
5050
5090
h
g f e
5190
5290
5310
5350
5430
d c b a
Z Y X W
o
5515
5545
5610
p q
5660
5680
5710
5780
r
5860
5934
6080
6190
6290
r
6320
q p o
6350
V U
6380
6490
s t u
6630
6670
6710
v
6740
6840
6885
6903
6966
7010
7130
7220
7280
7320
7372
7390
7480
7530
7570
7610
v u t
7650
7690
7745
s
T S R
7800
Q
7840
8020
8080
7910
P O N
8260
8320
8360
8440
8470
8570
8720
8800
8940
L
8990
9010
D C B A
9106
9130
9150
9173
Reliability of prediction:
<0.5
1.0
unpaired:
paired:

Supplementary Figure S3: The PPfold 3.1 structure colored by comparison with the Watts09 structure. The predictions were made with identical SHAPE data. Common structural components are indicated in red (base pairs) and blue (unpaired nucleotides). Different base pairing partners (but pairing in both structures) are indicated by yellow. Nucleotides paired in one but unpaired in the other are indicated in brown. 72% of all unpaired nucleotides and 31% of base paired nucleotides were found in both predictions. Over half of the genome is unpaired in both predictions (blue).

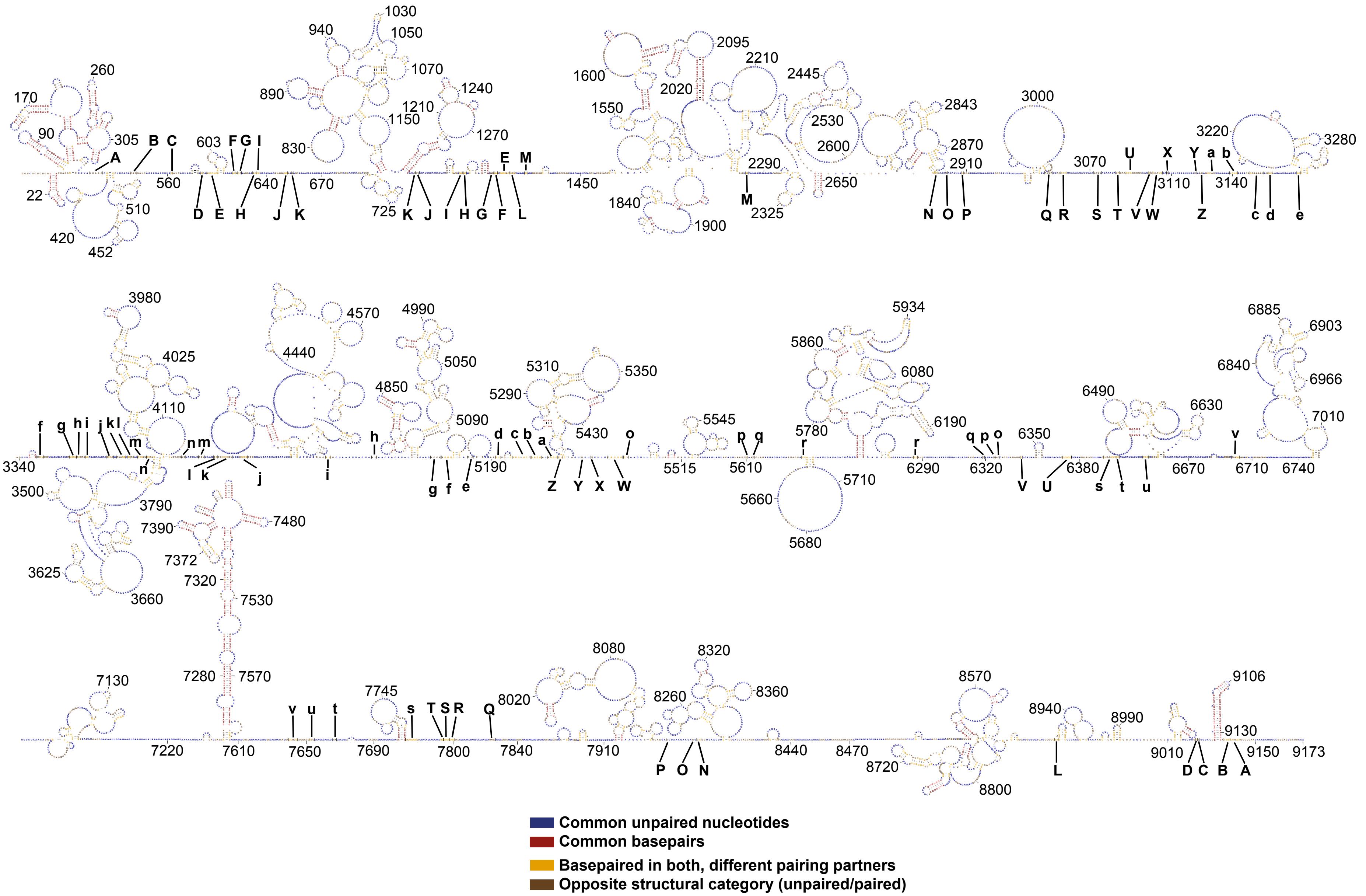
Common unpaired nucleotides
Common basepairs
Basepaired in both, different pairing partners
Opposite structural category (unpaired/paired)

Supplementary Figure S4: Phylogenetic analysis of the proposed structural model. The proportion of CBCs (two-sided covariation) is plotted. This is the proportion of base pairs consistent with the structural prediction (A-U, G-C, G-U), when removing gaps and the most frequent canonical base pair, as described in the Methods section.

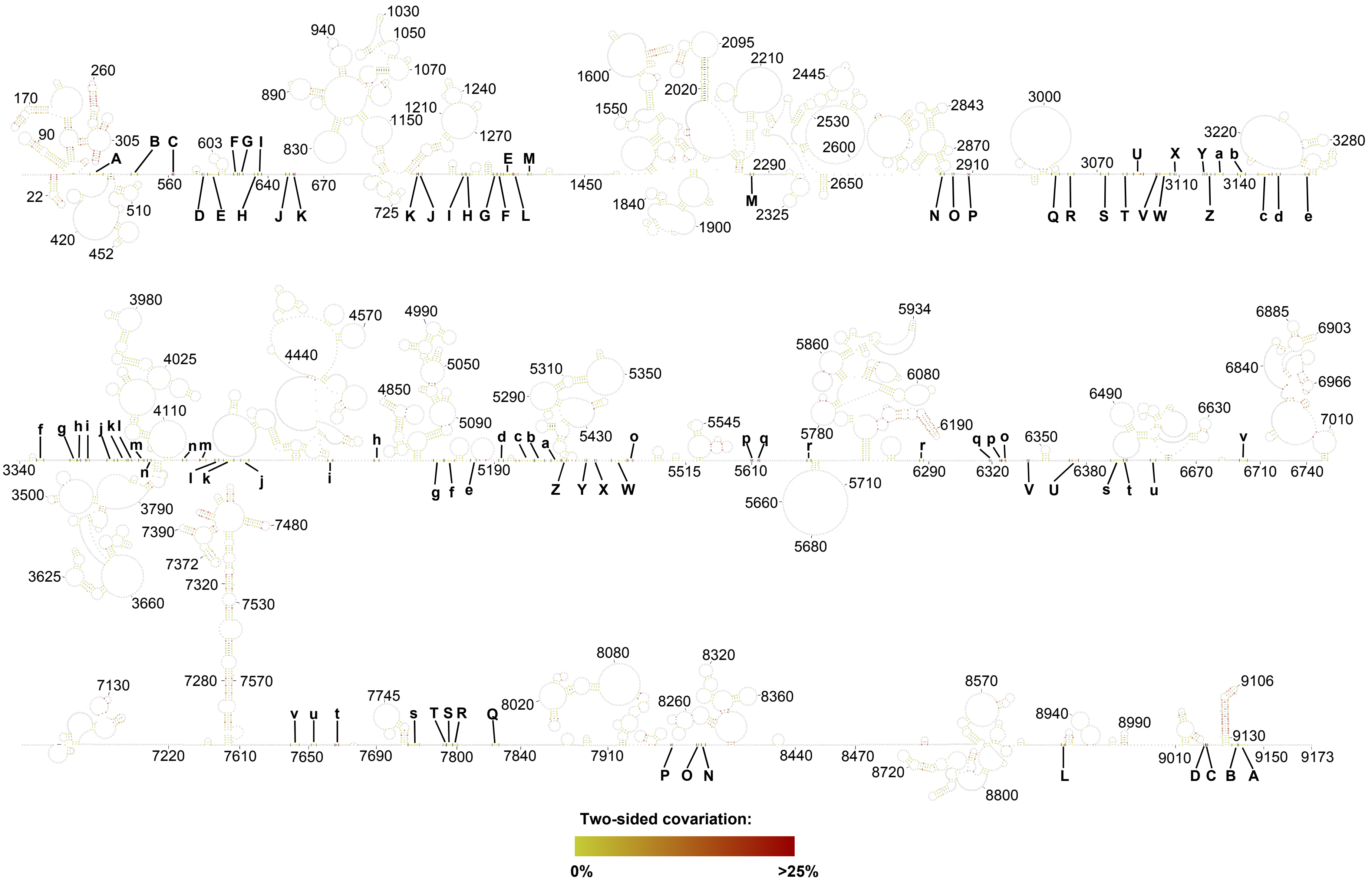

22
90
170
260
305
420
452
510
560
603
640
670
725
830
890
940
1030
1050
1070
1150
1210
1240
1270
1450
1550
1600
1840
1900
2020
2095
2210
2290
2325
2445
2530
2600
2650
2843
2870
2910
3000
3070
3110
3140
3220
3280
A B C D E F G H I J K L M N O P Q R S T U V W X Y Z a b c d e
3340
3500
3625
3660
3790
3980
4025
4110
4440
4570
4850
4990
5050
5090
5190
5290
5310
5350
5430
5515
5545
5610
5660
5680
5710
5780
5860
5934
6080
6190
6290
6320
6350
6380
6490
6630
6670
6710
6740
6840
6885
6903
6966
7010
f g h i j k l m n o p q r s t u v
7130
7220
7280
7320
7372
7390
7480
7530
7570
7610
7650
7690
7745
7800
7840
7910
8020
8080
8260
8320
8360
8440
8470
8570
8720
8800
8940
8990
9010
9106
9130
9150
9173
Two-sided covariation:
0%
>25%

Supplementary Figure S5: Analysis of sequence conservation along the proposed structural model.

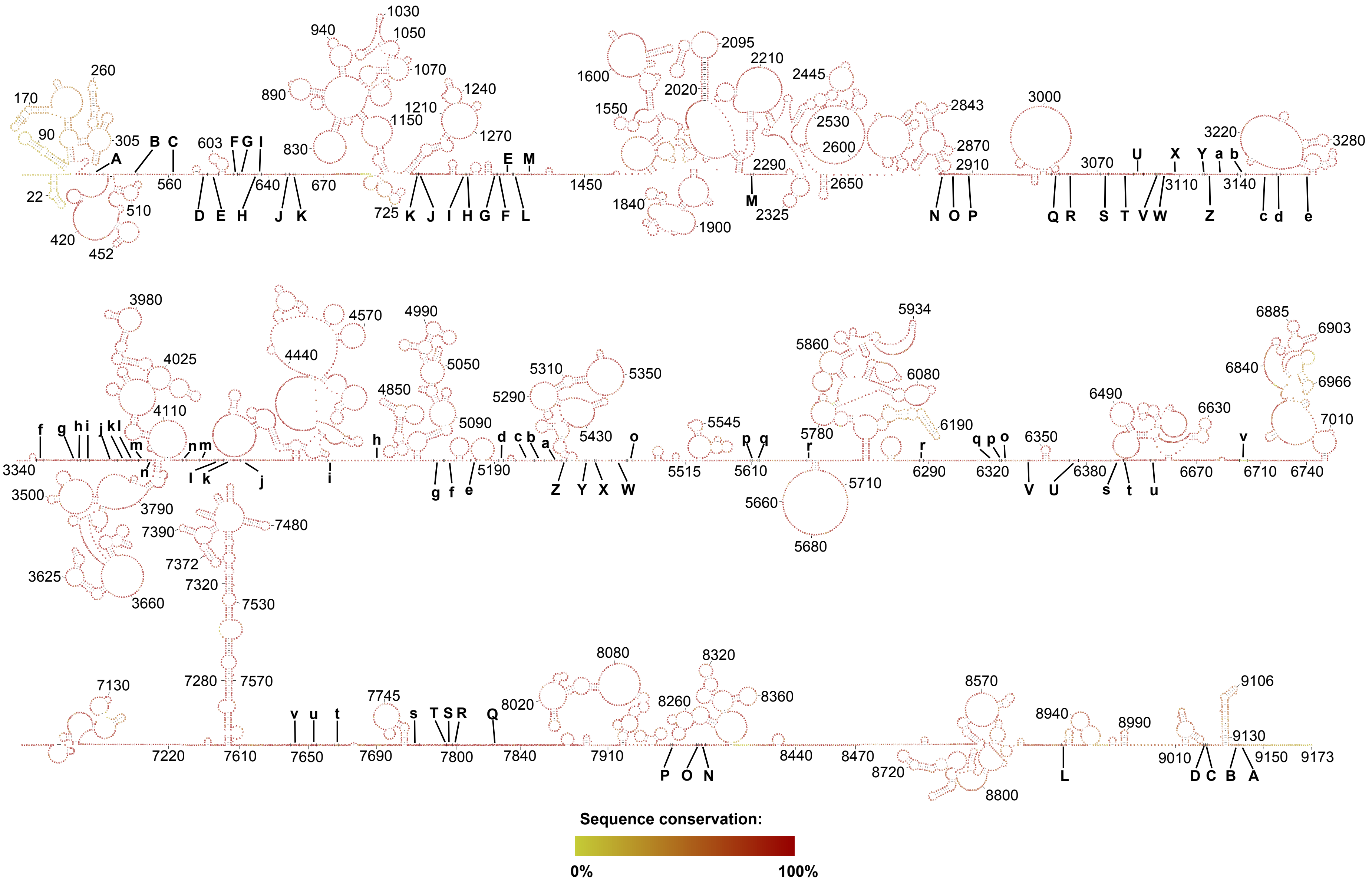

1030
1050
940
1070
260
890
1240
1210
2095
2210
1600
2445
2020
1550
2843
3000
170
1150
2530
90
305
A
B
C
603
F
G
I
830
1270
2600
2870
3220
3280
E
M
2290
2910
3070
U
X
Y
a
b
22
560
640
670
1450
2650
3110
3140
510
D
E
H
J
K
725
K
J
I
H
G
F
L
1840
M
2325
N
O
P
Q
R
S
T
V
W
Z
c
d
e
1900
420
452
3980
4570
4990
5934
6885
4440
6903
4025
5050
5860
5310
5350
6080
6840
6966
4850
5290
6490
6630
4110
5090
5545
6190
7010
f
g
h
i
j
k
l
m
n
m
h
d
c
b
a
5430
o
p
q
5780
r
r
q
p
o
6350
v
3340
5515
5610
6290
6320
6380
6670
6710
6740
n
l
k
j
i
g
f
e
5190
Z
Y
X
W
V
U
s
t
u
3500
5710
3790
5660
7480
7390
5680
7372
3625
7320
7530
3660
8080
8320
7130
7280
7570
9106
7745
8570
8260
8360
v
u
t
s
T
S
R
Q
8020
8940
8990
9130
7220
7610
7650
7690
7800
7840
7910
8440
8470
9010
9150
9173
8720
P
O
N
L
D
C
B
A
8800
Sequence conservation:
0%
100%

Supplementary Figure S6: The proposed structural model colored by SHAPE values.

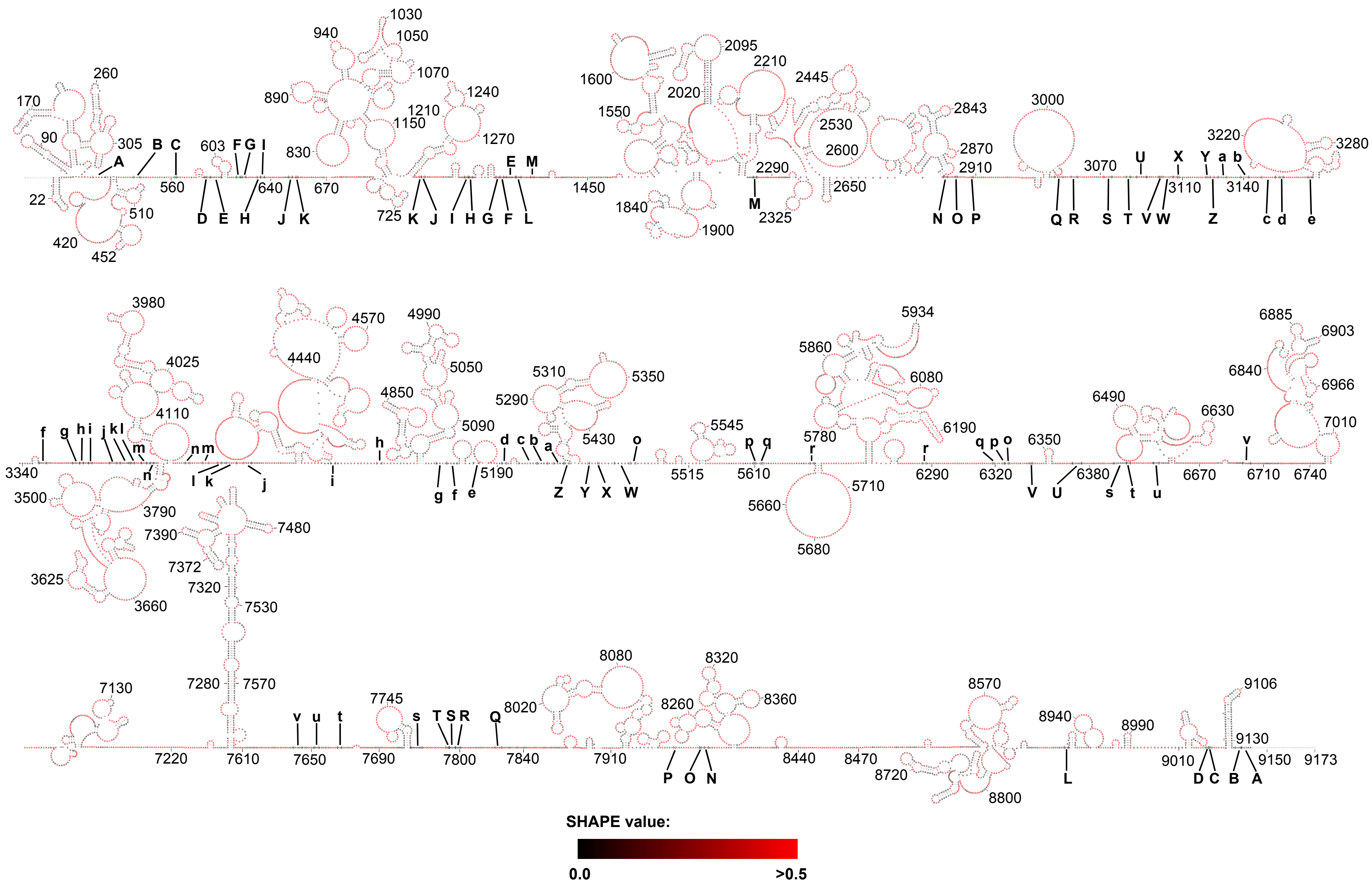
SHAPE value:
0.0
>0.5

Supplementary Figure S7: The Venn diagram illustrates the number of shared base pairs between the 3 predictions: Pollom2013, Watts09 and PPfold+SHAPE. The base pair overlap of the PPfold+SHAPE prediction to the Pollom2013 dataset is highly similar to the overlap with Watts09.

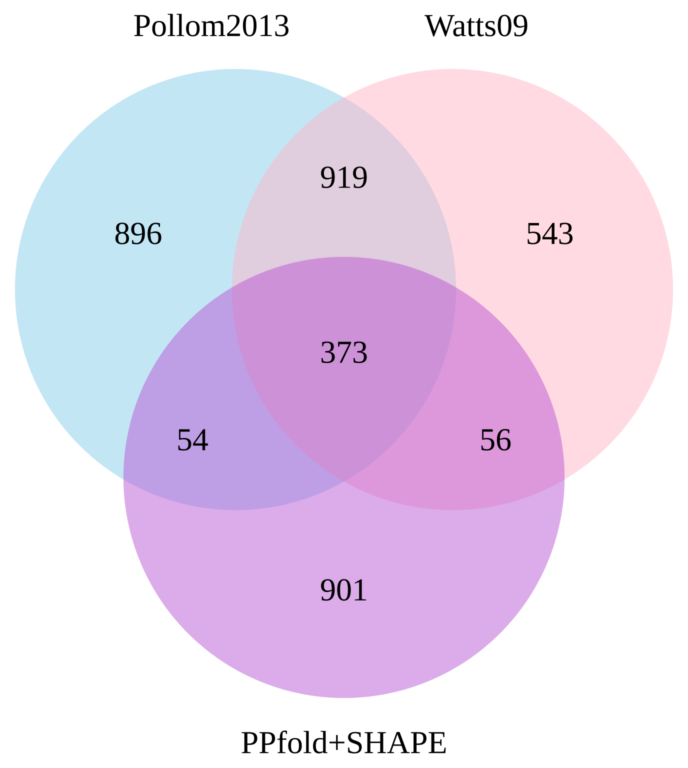
Pollom2013
Watts09
919
896
543
373
54
56
901
PPfold+SHAPE
