# Supplementary material for: Full-length RNA structure prediction of the HIV-1 genome reveals a conserved core domain
Source: Nucleic Acids Res. 2015 Oct 17;43(21):10168–79. doi: 10.1093/nar/gkv1039 (PMC4666355; doi:10.1093/nar/gkv1039)
Supplement: SUPPLEMENTARY DATA [file supp_gkv1039_nar-01265-r-2015-File009.pdf]

Supplementary Methods

Sequences in the 38-sequence HIV-1 alignment

The accession numbers of the 37 sequences are:

Ref.A1.AU.03.PS1044_Day0.DQ676872, Ref.A1.RW.92.92RW008.AB253421, Ref.A1.UG.92.92UG037.AB253429, Ref.A2.CD.97.97CDKTB48.AF286238, Ref.A2.CM.01.01CM_1445MV.GU201516, Ref.A2.CY.94.94CY017_41.AF286237, Ref.B.FR.83.HXB2_LAI_IIIB_BRU.K03455, Ref.B.NL.00.671_00T36.AY423387, Ref.B.TH.90.BK132.AY173951, Ref.B.US.98.1058_11.AY331295, Ref.C.BR.92.BR025_d.U52953, Ref.C.ET.86.ETH2220.U46016, Ref.C.IN.95.95IN21068.AF067155, Ref.C.ZA.04.04ZASK146.AY772699, Ref.D.CD.83.ELI.K03454, Ref.D.CM.01.01CM_4412HAL.AY371157, Ref.D.TZ.01.A280.AY253311, Ref.D.UG.94.94UG114.U88824, Ref.F1.BE.93.VI850.AF077336, Ref.F1.BR.93.93BR020_1.AF005494, Ref.F1.FI.93.FIN9363.AF075703, Ref.F1.FR.96.96FR_MP411.AJ249238, Ref.F2.CM.02.02CM_0016BBY.AY371158, Ref.F2.CM.95.95CM_MP255.AJ249236, Ref.F2.CM.95.95CM_MP257.AJ249237, Ref.F2.CM.97.CM53657.AF377956, Ref.H.BE.93.VI991.AF190127, Ref.H.BE.93.VI997.AF190128, Ref.H.CF.90.056.AF005496, Ref.H.GB.00.00GBAC4001.FJ711703, Ref.J.CD.97.J_97DC_KTB147.EF614151, Ref.J.CM.04.04CMU11421.GU237072, Ref.J.SE.93.SE9280_7887.AF082394, Ref.K.CD.97.97ZR_EQTB11.AJ249235, Ref.K.CM.96.96CM_MP535.AJ249239, NC_001802, AF324493

The 37-sequence alignment was extended with the “Watts09” sequence, which was originally published by Watts et al. (2009, Nature 460:711-716).

Reliability scores

Under the combined statistical model implemented in PPfold 3.1 (1), the probability of the secondary structure, $P(\sigma)$ is defined as:

$$P(\sigma) = \frac{P(\sigma|M_s)P(H|\sigma)P(D|\sigma, M_t)}{P(D, H|M_s, M_t)}$$

where:

- $P(\sigma|M_s)$ is the prior probability of the secondary structure σ, generated by the stochastic context-free grammar (SCFG) model M_s.
- $P(D|\sigma, M_t)$ is the likelihood of the input alignment D, given the evolutionary model M_t and the secondary structure σ.
- $P(H|\sigma)$ is the empirical likelihood of the SHAPE data, given the secondary structure secondary structure σ. We note that the SHAPE data are only assumed to depend on the local secondary structure, and not on nucleotide identity or sequence position. Furthermore, data values for nucleotides in the same pair are not correlated (1).
- $P(D, H|M_s, M_t)$ is a normalizing constant.

The “reliability score” for position is the probability of the structure prediction for the nucleotide in that position under this statistical model. The probability of a basepair between position *i* and *j* is thus the sum of the probabilities of all structures containing that basepair:

$$P_d(i,j) = \sum_{\sigma \in S_{i\sim j}} P(\sigma)$$

where $S_{i\sim j}$ is the set of all possible (nested) secondary structures for the sequence or alignment that contain a basepair between the positions *i* and *j*. The probability of a single-stranded nucleotide in position *i* is the probability that *i* does not participate in any basepair:

$$P_s(i) = 1 - \sum_{j \neq i} \sum_{\sigma \in S_{i\sim j}} P(\sigma)$$

If a basepair is predicted between *i* and *j*, the reliability score for both positions will be $P_d(i,j)$. If a single-stranded nucleotide is predicted at position *i*, the reliability score for this position will be $P_s(i)$. The values of $P_d(i,j)$ and $P_s(i)$are in practice computed using the inside-outside algorithm.

On an empirical basis, we consider a score above 0.8 to be “high reliability”, a score between 0.5 and 0.8 to be “medium reliability”, and a score of 0.5 or below to be “low reliability”. High scores are only possible when the SHAPE data and evolutionary data support each other. High reliabilities, therefore, suggest a robust prediction, whereas low reliability scores are associated with random errors.

Re-normalizing the SHAPE data

As noted in Table 1 of the paper, in prediction 13 we used PPfold 3.1 with re-normalized SHAPE data. Here we detail the rationale behind this, and the method used to re-normalize the data.

The published SHAPE counts had originally been normalized to a scale such that 1.0 represents the nucleotides with "highest reactivity" (2,3). The percentage of nucleotides with normalized SHAPE reactivities over 1.0 was 9% in the case of the ribosomal dataset (3), and 5% in the case of HIV-1 (2). However, the percentage of values over 1.0 is significantly higher in the case of unpaired nucleotides than in the case of paired nucleotides. This means that for a highly flexible sequence, normalizing to the highest reactive nucleotides will bias the normalized SHAPE reactivities towards lower values. In this method of normalization, the bias will be greater the more flexible the sequence is.

The true proportion of unpaired nucleotides in the HIV-1 genome, a, is unknown at the time of probing its structure. However, we expect that the SHAPE reactivities in HIV-1 will follow the distribution:

$$P(SHAPE\ value) = a \cdot P(SHAPE\ value|unpaired) + (1-a) \cdot P(SHAPE\ value|paired) \qquad \textbf{[1]}$$

The true proportion of unpaired unpaired nucleotides a can then be estimated using a quantile-to-quantile mapping between the observed distribution and this expected distribution. In the re-normalization, we scaled the observed SHAPE data such that the threshold for the top 5% of values matches that of **[1]**.

The distributions *P(SHAPE value|paired)* and *P(SHAPE value|unpaired)* were determined using SHAPE data measured on the *E. coli* 16S and 23S sequences, and are shown in Figure A (see also (4,5)).

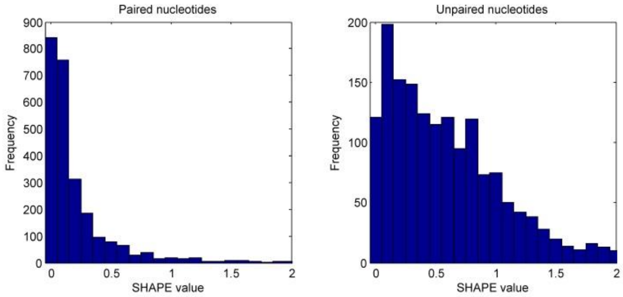

Figure A: The frequency distributions *P(SHAPE value|paired)* (left) and *P(SHAPE value|unpaired)* (right) from ribosomal data.

The distributions *P(SHAPE value)* are shown in Figure B, for the ribosome (left) and HIV-1 (right).

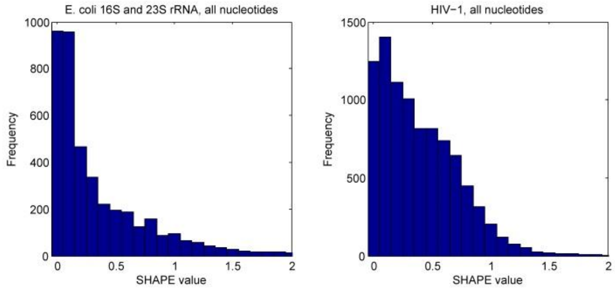

Figure B: The frequency distributions *P(SHAPE value)* for ribosomal data (left) and HIV-1 (right). It is clear that the HIV-1 genome is much more flexible than the ribosome.

We expect that that for the “correct” value of *a*, the distribution of observed SHAPE values is statistically indistinguishable from **[1]**. We estimated *a* by fitting: the empirical distribution of SHAPE values was subtracted from the theoretical distribution for various values of *a*, and the probability differences were plotted. The “best-fit” case is when the differences are least prominent.

As Figure C shows, the best-fit value was around 40% in the case of the *E. coli* ribosomal subunits. The true percentage of unpaired nucleotides in the *E. coli* ribosomal 16S and 23S rRNA taken together is 39.5%. The method, therefore, correctly recovered the true percentage of unpaired nucleotides in the *E. coli* ribosomal RNA.

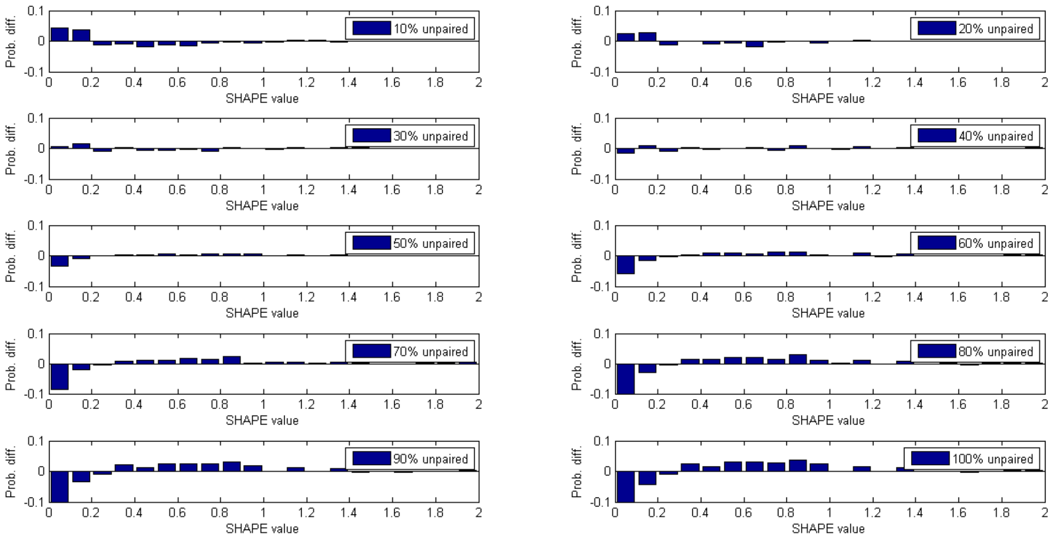

Figure C: Estimating the degree of basepairing in the E. coli 16S and 23S ribosomal subunits, as a confirmation of the method.

In the case of HIV-1, however, the “best-fit” case was above 80% unpaired nucleotides, as shown in Figure D. Re-normalizing to 80% unpaired nucleotides, we multiplied the published SHAPE values by 1.8. In this case, approximately 30% of values fall over 1.0.

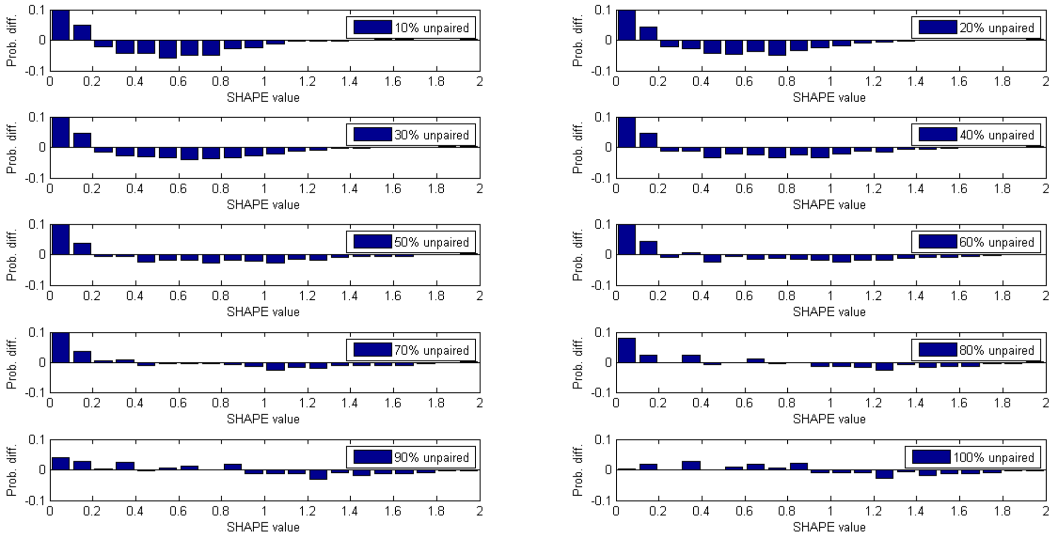

Figure D: Estimating the degree of basepairing in the HIV-1 genome.

1. Sukosd, Z., Knudsen, B., Kjems, J. and Pedersen, C.N. (2012) PPfold 3.0: fast RNA secondary structure prediction using phylogeny and auxiliary data. *Bioinformatics*, **28**, 2691-2692.
2. Watts, J.M., Dang, K.K., Gorelick, R.J., Leonard, C.W., Bess, J.W., Jr., Swanstrom, R., Burch, C.L. and Weeks, K.M. (2009) Architecture and secondary structure of an entire HIV-1 RNA genome. *Nature*, **460**, 711-716.
3. Deigan, K.E., Li, T.W., Mathews, D.H. and Weeks, K.M. (2009) Accurate SHAPE-directed RNA structure determination. *PNAS*, **106**, 97-102.
4. Sukosd, Z., Swenson, M.S., Kjems, J. and Heitsch, C.E. (2013) Evaluating the accuracy of SHAPE-directed RNA secondary structure predictions. *Nucleic Acids Res*, **41**, 2807-2816.
5. Eddy, S.R. (2014) Computational analysis of conserved RNA secondary structure in transcriptomes and genomes. *Annual review of biophysics*, **43**, 433-456.
